# Elemental Profiling for the Detection of Food Mixtures: A Proof of Principle Study on the Detection of Mixed Walnut Origins Using Measured and Calculated Data

**DOI:** 10.3390/molecules29143350

**Published:** 2024-07-17

**Authors:** Marie-Sophie Müller, Esra Erçetin, Lina Cvancar, Marie Oest, Markus Fischer

**Affiliations:** Hamburg School of Food Science, Institute of Food Chemistry, University of Hamburg, Grindelallee 117, 20146 Hamburg, Germany; mariesophie.mueller@uni-hamburg.de (M.-S.M.); esr.ercetin@gmail.com (E.E.); lina.cvancar@uni-hamburg.de (L.C.); marie.oest@uni-hamburg.de (M.O.)

**Keywords:** walnuts, element profiling, mixtures, inductively coupled plasma mass spectrometry, chemometrics

## Abstract

Element profiling is a powerful tool for detecting fraud related to claims of geographical origin. However, these methods must be continuously developed, as mixtures of different origins in particular offer great potential for adulteration. This study is a proof of principle to determine whether elemental profiling is suitable for detecting mixtures of the same food but from different origins and whether calculated data from walnut mixtures could help to reduce the measurement burden. The calculated data used in this study were generated based on measurements of authentic, unadulterated samples. Five different classification models and three regression models were applied in five different evaluation approaches to detect adulteration or even distinguish between adulteration levels (10% to 90%). To validate the method, 270 mixtures of walnuts from different origins were analyzed using inductively coupled plasma mass spectrometry (ICP-MS). Depending on the evaluation approach, different characteristics were observed in mixtures when comparing the calculated and measured data. Based on the measured data, it was possible to detect admixtures with an accuracy of 100%, even at low levels of adulteration (20%), depending on the country. However, calculated data can only contribute to the detection of adulterated walnut samples in exceptional cases.

## 1. Introduction

Element profiling is a growing research area, describing the analysis of elements and isotopes and their ratios. This omics discipline, also known as isotopolomics, is suitable for determining the origin of plant-based foods due to the correlation between the elemental composition of a food and the soil in which it was grown [1,2,3,4].

Consumers are willing to pay higher prices for high-quality food, e.g., for certain varieties and origins, but also for certain production methods [5]. Walnuts generally belong to these high-quality foods, as they are said to have health-promoting properties [6]. They are also one of the major tree nut crops. In 2020, the worldwide production volume was 2.3 Mio tons, with China and the US accounting for over 75% of the total [7,8]. Walnuts from certain regions of origin in France are also labelled with a “Protected Designation of Origin”, which leads to higher prices and at the same time provides an incentive for economically motivated food fraud [9]. The complexity of the global production chain offers additional opportunities for misdeclaration, which are often difficult to verify. Therefore, the ability to trace the origin of food using analytical methods is an essential tool and a current research topic to counteract food fraud [10,11,12,13,14]. The detection of adulteration in mixtures is more difficult than the identification of pure samples, as the differences detected between the two samples become less clear due to the mixture. From the counterfeiter’s point of view, mixing samples would be much more efficient in disguising a counterfeit. The European Union already works on regulations for the declaration of foods containing the same raw material but of different origins, e.g., honey or nuts [15]. Although many successful studies have been conducted to determine the origin of foods, there is a lack of studies analyzing mixtures of the same food. Nevertheless, the development of methods to detect mixtures of different origins is becoming increasingly important.

In a recent study, the origin of walnuts was analyzed by determining the concentration of 47 elements with inductively coupled plasma mass spectrometry (ICP-MS). Segelke et al. [16] studied the origin of walnuts from ten different countries and three different harvest years. The origin could be determined with an overall accuracy of 73% for unadulterated samples [16].

Analyzing nuts with ICP-MS is challenging due to the low element concentration in high-fat foods [14,16,17,18,19,20]. The differentiation of origin is based on the differences in element concentrations. Thus, when analyzing mixtures of the same food, the observed differences in element concentrations are further attenuated, which requires the use of highly sensitive instrumentation. ICP-MS offers the lowest limits of detection for element concentrations down to parts per trillion (ppt-ng/L) and thus the possibility to identify mixtures of different origins [1,21]. To our knowledge, there is no other approach used to analyze walnut mixtures of different origins based on the element profile. To date, the element profile has only been used for detecting mixtures of different foods [22,23,24,25].

The aim of this study is to develop a method for the detection of walnut mixtures from six different origins in nine different mixing ratios. To test the model, walnut mixtures of different origins and different ratios were measured with ICP-MS. The measurement effort increases rapidly with different mixing ratios and different sample origins. Therefore, it was additionally tested whether calculated mixtures can reduce the experimental effort by using data of pure walnut samples analyzed in a previous study using ICP-MS [16]. A similar work attempted to identify hazelnut mixtures using nuclear magnetic resonance spectroscopy, showing promising results for correlation between calculated and measured samples [26]. However, matrix effects and differences in instrument conditions can complicate the comparison. A major advantage of element profiling is its superior stability in relation to analyte signals compared to other analyte groups, such as metabolites [17,21].

This study serves as proof of principle that mixtures containing the same food of different origins can be detected via ICP-MS. Additionally, it was studied whether measured element concentrations can be compared with calculated element concentrations and potentially support them in authentication approaches. This would reduce the use of hazardous chemicals and thus promote more resource-efficient analysis. Different multivariate data analysis methods were used to evaluate which method provides the best results for discriminating walnut mixtures of different origins by comparing calculated and measured data.

## 2. Results and Discussion

**Figure 1:**
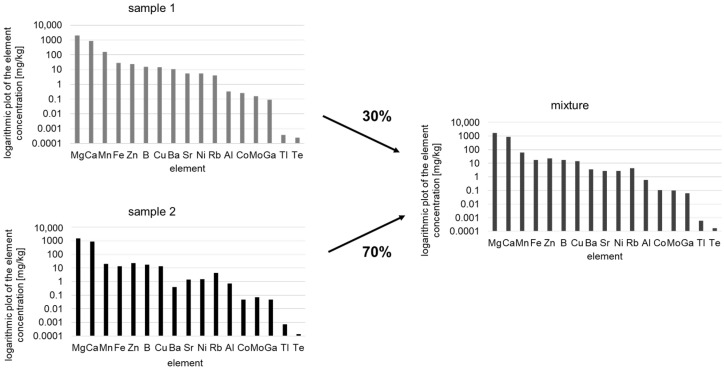
Schematic presentation of two samples (sample 1 and sample 2) mixed with each other in a ratio of 70% to 30%. The right picture representing the mixture shows calculated differences in element concentrations (logarithmic plot of the element concentration).

Element profiling using ICP-MS or IR-MS is frequently used to authenticate foodstuffs. Current research refers, for example, to the differentiation of different origins, whereby mixtures of origins have hardly been investigated to date [23,24]. Nevertheless, it becomes more important to analyze mixtures as well. In the present study, the adulteration of walnuts mixtures of different origins was investigated. This type of analysis is not about the misdeclaration of an entire batch but rather about the detection of “cheap” admixtures of high-quality goods for the purpose of profit maximization—a typical case of financially motivated food fraud. Analyzing mixtures is very difficult due to the large number of possible sample combinations in different mixing ratios. Furthermore, element profiling is limited to the detection of different concentrations of the same element pattern when different geographical origins are distinguished. The analysis of mixtures of different origins is even more challenging than the characterization of single origins, as mixing samples of different origins only leads to small variations in element concentrations compared to the pure samples (Figure 1).

A previous study using ICP-MS showed promising results for the classification of authentic, unadulterated walnuts from ten different countries [16]. Based on the same data set, the sample size was reduced for technical reasons (to have a more balanced number of samples for each country), using only six countries with at least fifteen samples each, resulting in a sample size of 206 walnuts. A random subset of these samples was used to prepare walnut mixtures using samples from different origins in different mixing ratios. The mixtures were digested and measured via ICP-MS and afterwards evaluated by internal and external calibration. The amount of each element was given in mg/kg. For the model evaluation, only the significant elements for origin discrimination determined in a previous study were used for origin differentiation [16]. Due to the immense increase in the amount of work involved in preparation and measurement, it was examined whether some of the measurements could be replaced with simulations. Using 206 walnut samples, a total of 21.115 different combinations of samples are possible for one mixing ratio. After eliminating all mixture combinations containing samples from the same country, 16.783 combinations remain. If nine different mixing ratios (from 10% to 90% in 10% steps) are used for the calculation, this results in over 1.5 × 10^5^ possible sample mixtures (for calculation, see Table S1). Breaking down and measuring all these sample combinations is a time-consuming and costly process. By calculating possible sample mixtures and measuring a subset of the mixtures, the measurement effort can be significantly reduced. This proof of principle examines whether measured walnut mixtures can be detected based on their element profile. Additionally, it was investigated whether measured data is comparable with calculated data. If so, only a small validation set would need to be measured to confirm the correlation. This would simplify the workflow and make the method more attractive for routine analysis.

### 2.1. Data Calculation

For model creation, the element concentrations of each possible mixture were calculated. The calculation was performed using a linear combination of element concentrations (Equation (1)).

(1) element concentration of mixture=(x·element concentration of sample 1) + (y·element concentration of sample 2)

The variables *x* and *y* in this formula are the respective ratios of the mixtures (sample set A = calculated partial quantity; sample set B = experimentally measured partial quantity). All results are given in mg/kg. Sample set B (measured mixtures) was mixed in the particular ratio to approximately 500 mg and then digested according to Section 3.2. In this way, the total element concentrations for both sample sets were determined. Triplicate determinations of sample set B (measured mixtures) yielded reproducible values for the 17 elements analyzed. Furthermore, individual measurements of the samples were performed and the results of the triplicate analysis were averaged.

### 2.2. Deviation of Measured and Calculated Data for Mixtures

**Figure 2:**
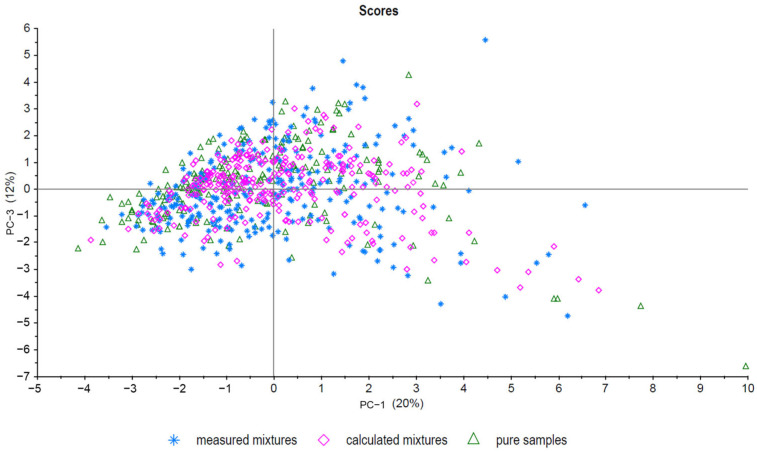
PCA of all pure samples (triangle), all calculated mixtures (diamond, sample set A), and all measured mixtures (star, sample set B). PC-1 plotted against PC-3.

**Figure 3:**
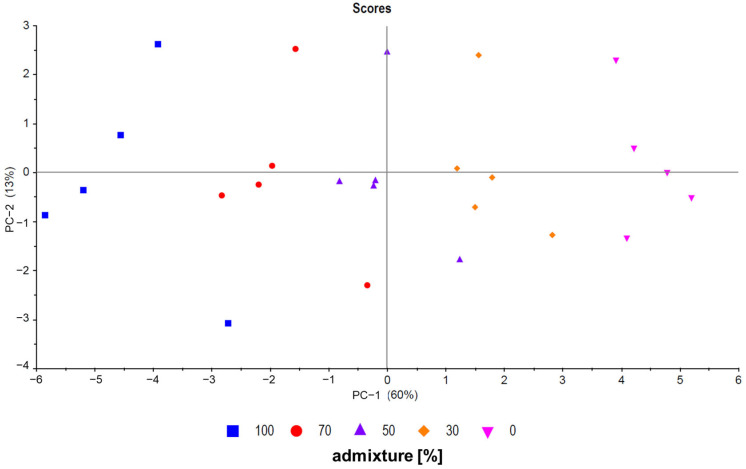
PCA showing five French samples (inverted triangle), five Chinese samples (box), and three different mixture ratios (dot, triangle, and diamond) for each of the five samples (PC-1 plotted against PC-2, sample set B (measured mixtures)).

Principle component analysis (PCA) is a useful unsupervised tool to visualize data and was used for model evaluation [27]. First, all pure samples (sample set C), all measured mixtures (sample set B), and all calculated mixtures (sample set A) were analyzed. As expected, there was an overlap between all three sample groups, as shown in Figure 2 (PC-1 plotted against PC-3). No evident separation between sample set A and sample set B was observed. In addition, sample set C (pure samples) forms a cluster with the other groups. The scattering of the samples is also comparable between the groups. Figure 3 shows a PCA (PC-1 plotted against PC-2) of five exemplary measured mixtures (ratios: 100/0, 70/30, 50/50, 30/70, and 0/100) of five different French and Chinese samples each. The mixed samples are located between the pure samples, showing a trend from negative values to positive values for PC-1 with descending admixture. Even though PCA is a useful screening tool to visualize and analyze data quickly, classification and regression models provide higher accuracy in detecting mixtures. Five different approaches were used to detect mixtures and compare measured and calculated data. The next section gives an overview of the results of the different methods used to detect and/or quantify the admixtures in the walnut samples.

### 2.3. Classification and Regression Models

In order to obtain an overview of the initial situation of the data, a six-class classification model was created in the first step which contained all the countries included in this study. The data of sample set C (pure samples) were used to create the model. In addition to various classification methods, different methods for data pretreatment were also tested. Detailed information is shown in the classification models section of Table 1. The model was validated using a repeated cross validation (5 folds, 5 repeats).

**Figure 4:**
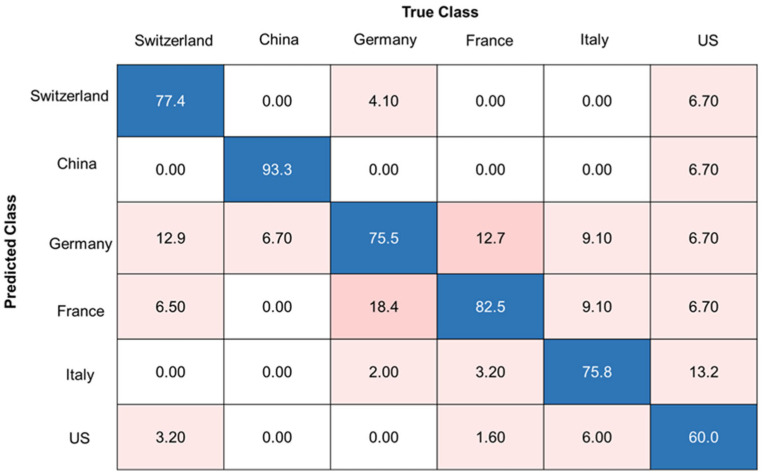
Confusion matrix of the best classification model (SVM) for pure walnut samples (sample set C), accuracies are given in %, overall accuracy of 77.5%, data pretreatment: decadic logarithm. Blue boxes highlighting correct classifications, pink boxes highlighting false classifications.

The best classification accuracy in the prediction of the pure samples was achieved with the SVM (radial kernel) model and a decadic logarithm as the data pretreatment method, resulting in an overall accuracy of 77.5%. The corresponding confusion matrix can be found in Figure 4. It can be observed that the samples from the US are the blind spot of the model. At least one sample was classified as a sample from each of the other countries. Distinguishing between Germany and France was also difficult, resulting in most of the misclassifications between the two countries. Most misclassifications occurred between countries that are in close proximity. An overview of all classification models and their results can be found in Table S2. As discussed in the following sections, we tested whether the classification accuracy of 77.5% could be improved by trying different two-class classifiers (approach II and III, Table 2).

In the case of food fraud, the most important thing is to know whether it is counterfeit. Only secondary is the question of where the counterfeit product comes from. In addition, it was investigated whether the detection of mixtures is possible by using the data of the measured mixtures (sample set B) and calculated mixtures (sample set A) in different classification and regression models (see Table 2).

#### 2.3.1. Approach I

The first approach that attempted to identify walnut mixtures used one-class classification models. Previous studies using spectroscopy (e.g., infrared or Raman spectroscopy) have shown that one-class classifiers are a powerful tool to detect adulteration in food [34,35,36,37,38]. OC-SVM and OC-SIMCA models and different data pretreatment methods were applied in this study (Table 1, one-class classification). The model parameters can be found in Table S3. A 4-fold cross validation was carried out for both models. These approaches require only one class for model creation, this being in the present approach the pure samples (sample set C). A one-class model was created for each country. An independent test set was used for classification. If the samples in the test set are related to the samples used for model building, they are classified as the same class. If this is not the case, they are identified as outliers. In this study, the test set contains the adulterated samples of the respective country in all adulteration levels. If the adulterated samples (mixtures) were classified as outliers, the classification was successful [39]. The approach was performed with both calculated mixtures (sample set A) and measured mixtures (sample set B) as individual test sets. Table 3 shows the best results for the OC-SVM and OC-SIMCA models.

The OC-SVM models showed good results for the prediction of the measured mixtures (sample set B), up to 98.9% for the Swiss model. The accuracies for the calculated mixtures (sample set A) were poorer, such that no comparison could be made between the calculated and the measured data. Although the classification accuracies for the measured mixtures showed high accuracies (up to 98.9%), the unadulterated pure samples (sample set C) had a low accuracy (down to 46.7%). Therefore, the detection of unadulterated walnut samples with the OC-SVM model is not suitable and, consequently, the high accuracies for the measured mixtures are questionable.

In comparison to the OC-SVM models, the prediction of pure samples (sample set C) with the OC-SIMCA models achieved better accuracies, with values of up to 100% for calibration and 86% for the validation set. In contrast to the SVM model, the accuracies for the detection of measured mixtures (sample set B) are weaker. An exception is the Swiss model, where sample set B (measured mixtures) can be recognized with an accuracy of 81.1%. The accuracies for the measured mixtures (sample set B) of the other countries vary from about 37% to 65%. The results for the calculated mixtures (sample set A) are worse than the results of the measured mixtures (sample set B). Further approaches were carried out during the study. Two-class classification models were tested to improve the performance of the models.

Take-home message I: One-class classifier approaches did not show satisfactory results for elemental data. Additionally, the comparability between the calculated and measured mixtures was not given, regardless of the models used.

#### 2.3.2. Approach II

Approach II works with two-class classification models, comparing one country to the corresponding mixtures of the respective country. Three different classification models and four data pretreatment methods were tested (Table 1, classification models). The models were validated using a repeated cross validation (5 folds, 5 repeats). The model parameters can be found in Table S3. One two-class model per country was created. One class contains the pure samples of the respective country (sample set C) and the other class contains either the measured or the calculated mixtures of the particular country. The test set contains samples of the two groups as well. If a pure or an adulterated sample was assigned to the corresponding class, the classification was successful. The approach was performed individually with sample set A (calculated mixtures) as well as with sample set B (measured mixtures). To check if the measured and calculated mixtures were comparable, measured mixtures were also used as an independent test set classified with the model containing calculated mixtures. All degrees of adulteration were considered at once in comparison to the pure samples. If the measured mixtures were allocated to the calculated mixtures, the validation was successful [40].

**Figure 5:**
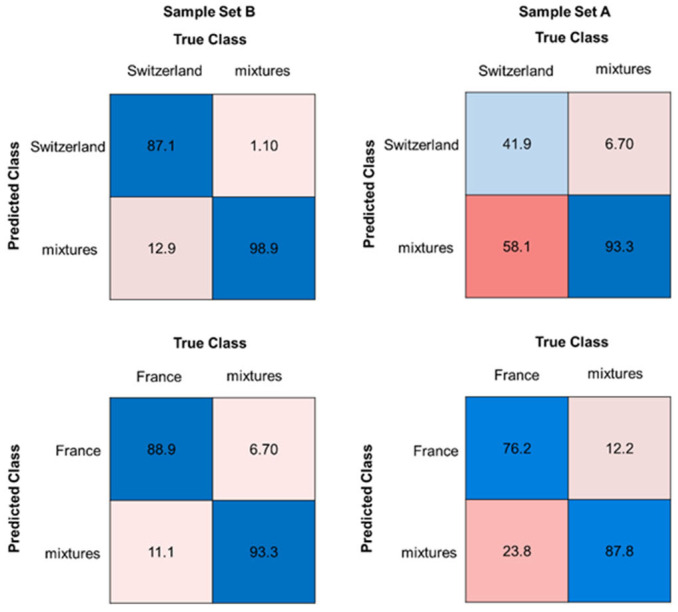
Confusion matrices for the classification models (RF) of Swiss and French walnut samples, each in comparison with all associated walnut mixtures, showing the accuracy in %. The left side shows sample set B (measured mixtures), the right side sample set A (calculated mixtures). Blue boxes highlighting correct classifications, pink to red boxes highlighting false classifications.

Random forest classification models showed the best accuracies. Table 3 shows an overview of the best results of each country. The overall accuracy for the models with sample set B (measured mixtures) showed very good results, ranging from 85.3% for the Italian model to 95.6% for the Swiss model. The corresponding confusion matrices for the Swiss and the French models are shown in Figure 5. The Swiss and the French models with sample set B (measured mixtures) showed good sensitivities for the pure samples (sample set C) and the measured mixtures. French and Swiss walnuts are among the more expensive ones, so a very good classification accuracy for these countries is important (accuracy of the French model was 91.3%) [41]. The classification models of the other countries also showed good sensitivity for the measured mixtures. Therefore, approach II is suitable for mixture detection.

Compared to sample set B (measured mixtures), the accuracies for models with sample set A (calculated mixtures) vary between 68.7% for the German model and 85.7% for the American model (Figure 5 and Figure S1). The models with the calculated mixtures (sample set A) showed also poorer sensitivity, especially for sample set C (pure samples). A similar behavior was observed for the models of the other countries, down to sensitivities of 0% for the calculated mixtures (Figure S1). It can be observed that sample set B (measured mixtures) shows up to 18% points (%pt) better accuracies than sample set A (calculated mixtures). Thus, as in approach I, there was no consistency between the measured and the calculated mixtures, but models with measured mixtures can be used for mixture recognition.

Additionally, it was checked whether the calculated mixtures (sample set A) could be validated by predicting measured mixtures (sample set B) as an independent test set. Therefore, the random forest models of the first variant, which contained the calculated mixtures, were used as training set. A repeated cross validation (5 folds, 5 repeats) was performed on the training set. Table 3 shows the results for the training set and the test set. Although the accuracy for the training set varied greatly depending on the country (from 65.5% to 85.7%), sample set B (measured mixtures) showed good predicting results (from 81.3% to 100%).

Take-home message II: The classification models using measured mixtures delivered very good results of up to 95.6% in detecting admixtures. So far, this approach showed the most promising results, offering the chance to predict measured mixtures using elemental data. The agreement between sample set A (calculated mixtures) and B (measured mixtures) is still not completely given with this approach, but the difference between the measured and the calculated mixtures is smaller compared to the first approach using one-class classifiers.

#### 2.3.3. Approach III

**Figure 6:**
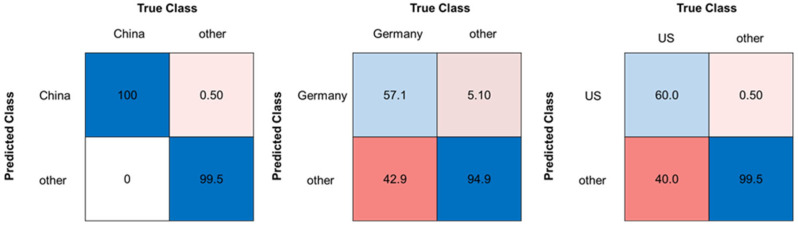
Confusion matrices for the classification models (SVM) of Chinese, German, and US walnut samples, each in comparison with all other pure walnut samples (sample set C), accuracies given in %. Blue boxes highlighting correct classifications, pink to red boxes highlighting false classifications.

Furthermore, a third approach was evaluated using two-class classifiers, comparing one country to all other countries as one group. A model was created for each country [17,26]. Analogous to the six-class classifier (see Section 2.3), the pure samples (sample set C) were used for model creation and the same methods as listed in Table 1 (section classification models) and data pretreatment were applied. The models were validated using a repeated cross validation (5 folds, 5 repeats). The best classification accuracies for the two-class-classifier were obtained using the SVM (radial kernel) model and a decadic logarithm as the data pretreatment method. The results for every country and the model parameters can be found in Tables S3 and S4. As expected, the overall accuracies improved compared to the six-class classifier (see Figure 4 and Figure 6). The model comparing China with the other countries showed the best performance, with an overall accuracy of 99.3%. The French and German models showed the lowest accuracies (German model: 85.6%, French model: 88.4%). The model for the US showed a good overall accuracy (96.7%), but only 60.0% of the samples from the US were classified as the true origin. This results in a weaker sensitivity of the three models compared to the other models. In relation to the second approach, the sensitivity for the Chinese, Italian, and American pure samples increased. The confusion matrices of the Chinese, German, and the US model can be seen in Figure 6. Further confusion matrices of the other countries can be found in Figure S2.

The measured data as well as the calculated data for the mixtures, including the respective country, were then predicted as individual test sets with the respective models. If an adulterated sample was classified as a sample from another country, the classification was successful. Two variants were tested. In the first variant, all adulteration levels were predicted together, as in approach II. In variant two, each adulteration level was predicted individually, evaluating from which level the adulteration could be detected. Table 3 shows the results for the first variant predicting all adulteration levels simultaneously. The results for the second variant can be found in Table 4 and Table S5. The first variant showed very good results of up to 95.6% for the Swiss and 80.2% for the French model, as in approach II with measured mixtures (sample set B). The other models also showed good accuracies, with the exception of the Chinese and Italian models (both 63%). The second variant shows that the measured mixtures (sample set B) were already well classified at low adulteration levels (Table 4 and Table S5). From a degree of adulteration of 50%, at least 70% of the measured mixtures were assigned to a different country. Some models showed even better accuracies at lower adulteration levels. At 20% adulteration, the Swiss model already showed 100% accuracy. Consequently, approach III is also suitable for the detection of measured mixtures (sample set B) on the basis of element data.

Looking at the comparability of the measured and calculated data, the German and the Italian models showed high conformity between sample set A (calculated mixtures) and B (measured mixtures) in both variants. In the second variant, the overall conformity ranged between 72.0% for the Swiss model and 92.0% for the German model. The largest discrepancy between the measured and calculated mixtures, which occurred in the Swiss model, can be explained by differences in the accuracy between the measured and calculated mixtures in the low adulteration levels (10–40%). From an adulteration level of 60%, all adulterated samples were classified correctly (Table S5). Overall, most classification models showed only a 10% points difference between the measured and calculated data from a degree of adulteration of 60%. Therefore, approach III showed the best comparability between the calculated and measured data so far.

Take-home message III: Measured mixtures can be detected with overall good classification accuracies using approach III. When looking at each adulteration level individually, even low adulteration levels can be detected depending on the country. So far, approach III showed the best conformity between the measured and calculated mixtures using the second variant.

#### 2.3.4. Approach IV and V

In addition, a totally different attempt was carried out, using classification models (approach IV) and regression models (approach V) based on calculated mixture data (sample set A) instead of the pure samples (sample set C). The training set contained the calculated mixtures in all adulteration levels, resulting in a nine-class classification or regression model. The calculated mixtures containing the same samples as the measured mixtures were not used for model creation to avoid overfitting. For this purpose, the models listed in the classification and regression models sections of Table 1 were used. The adulteration levels of both the measured (sample set B) and calculated data (sample set A) were again used as individual test sets. The best results for both classification and regression models were obtained with random forest models for the French samples. Due to the large amount of data, the model was validated with repeated cross validation with 10 folds and 5 repeats. The use of data pretreatment did not improve the results of the different models compared to the models without data pretreatment. As expected, the results of the test set with the calculated mixtures were significantly better than the results of the measured mixtures because the model was created with calculated data. The best accuracy for the measured French mixtures was 50%, while the best accuracy for the calculated French mixtures was 100%. No trend was observed, indicating that higher adulteration levels yielded better results (Figures S3 and S4). The regression models were evaluated with the root mean squared error (RMSE) and the coefficient of determination (R^2^). Even for the random forest regression model, the RSME was quite high (5.11%). For such large data sets with so many calculated mixtures, more significant variables would be beneficial for discrimination [42,43]. Isotopolomic data sets usually do not have so many significant variables, so a combination with stable isotope ratios in approach IV and V would be suitable [44].

Take-home message IV: To summarize, these models show no comparability between measured and calculated mixtures, as shown in the example for the French classification model in Table S6 and for the regression model in Table S7. Compared to this, the other approaches, which work with smaller data sets, showed better results. The bottom line is that the two approaches IV and V require too much effort.

#### 2.3.5. Comparison of Approaches I–V

**Figure 7:**
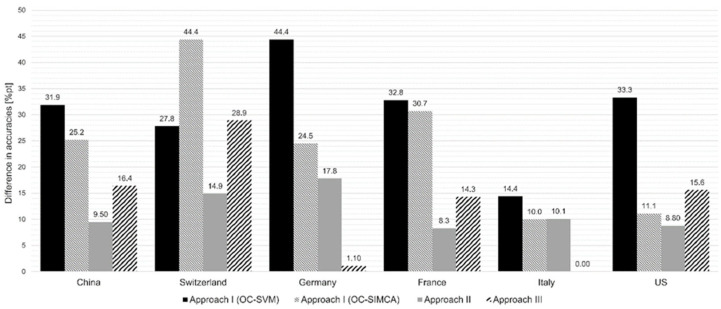
Comparison of the different approaches (I–III), showing the differences in the accuracies between the calculated (sample set A) and measured mixtures (sample set B).

Looking at the evaluation approaches (see Figure 7), it can be summarized

- that when working with elemental data, the one-class classifier (approach I) showed poorer results than the two-class classifier used in the second and third approaches.
- that the second and the third approaches deliver comparable results on average and are suitable to detect measured mixtures.
- that approach II showed the greatest agreement for the Chinese, Swiss, French, and American mixtures.
- that approach III shows the smallest differences between the measured and calculated mixtures when looking at the Italian and German mixtures.
- that the two approaches IV and V are ultimately too time-consuming and are therefore not recommended.

## 3. Materials and Methods

### 3.1. Reagents and Materials

Nitric acid (HNO_3_, ROTIPURAN^®^Supra, 69%) was acquired from Carl Roth GmbH & Co. KG (Karlsruhe, Germany) and hydrogen peroxide (H_2_O_2_, suprapur^®^, 30%) from Merck KGaA (Darmstadt, Germany). Ultrapure water (>18 MΩ) was received from a Direct Q purifying system (Merck Millipore Inc., Billerica, MA, USA). For external calibration, a multi-element standard solution was obtained from Merck KGaA (Darmstadt, Germany) containing 10 mg/L Li, Na, Mg, Al, K, V, Cr, Mn, Co, Ni, Cu, Ga, Rb, Sr, Mo, Ag, Cd, Te, Ba, Tl, Pb, Bi, U, 100 mg/L Be, B, Fe, Zn, As, Se, and 1000 mg/L Ca. As internal standards, single-element solutions (1 g/L Ge, Rh, In and Re) were purchased from Inorganic Ventures Inc. (Christiansburg, VA, USA). Different solutions for instrument tuning were received from Merck KGaA (Darmstadt, Germany) and PerkinElmer Inc. (Waltham, MA, USA). For detailed information, see Table S8 (Supporting Information). Argon (99.999%) was acquired from Sauerstoffwerk Steinfurt E. Howe GmbH & Co. KG (Steinfurt, Germany). All tubes and pipette tips used were steeped in nitric acid (3%, *v*/*v*) for 24 h and then flushed with ultrapure water and dried overnight before application.

### 3.2. Sample Preparation

A total of 206 walnut samples from six different countries (Germany (DE, 49 samples), Italy (IT, 33 samples), Switzerland (CH, 31 samples), France (FR, 63 samples), China (CN, 15 samples), and the United States of America (US, 15 samples)) and three harvest years (2017–2019) were used in this study, reducing the number of countries in comparison to the previous study (see Section 2) [16]. Sample preparation and digestion was handled in accordance with the methods of Segelke et al. [16]. Sample digestion was performed with an Ethos.lab microwave (MLS GmbH, Leutkirch, Germany). For method development, 270 walnut mixtures were prepared for measurement. From the 206 samples, a subset of samples was randomly selected to ensure an even distribution for each country. Nine different mixing ratios (90/10, 80/20, 70/30, 60/40, 50/50, 40/60, 30/70, 20/80, and 10/90) were prepared. For further evaluation, 540 mixtures were considered, as the 270 walnut mixtures described have both a 90/10 ratio for one country and a 10/90 ratio for the other. This results in ten mixtures per country and mixing ratio. Approximately 500 mg of each mixture was directly prepared in teflon vessels used for digestion. To each teflon vessel, 4 mL of nitric acid (69%) and 1 mL of hydrogen peroxide (30%) were added. During sample digestion, the mixtures were heated up to 200 °C within 16 min and then the temperature was held at 200 °C for further 20 min. After cooling, the digested samples were transferred to pre-cleaned tubes and then diluted with ultrapure water to 10 mL. Subsequently, the mixtures were measured by ICP-MS. One walnut mixture (CN/FR, 70/30) was used as a quality control sample (QC-sample) included in each digestion batch. The need for multiple determinations and the reproducibility of the measurement were evaluated during method optimization by measuring 30 mixtures in a triplicate analysis. A high-fat reference material (DLA ptSU08, DLA—Proficiency Tests GmbH, Oering, Germany) was used for verification of the method, showing good agreement with the high-fat matrix of walnuts. The results of the reference material can be found in Table S9.

### 3.3. Analytical Procedure

An Element 2 ICP-MS (Thermo Fisher Scientific Inc., Waltham, MA, USA) with a sector field mass analyzer in combination with an SC-E4 Autosampler (Element Scientific Inc., Omaha, NE, USA) was used for the multi-element analyses. Segelke et al. [16] determined 17 elements to be significant for the geographic origin of walnuts: Al, B, Ba, Ca, Co, Cu, Fe, Ga, Mg, Mn, Mo, Ni, Rb, Sr, Te, Tl, and Zn. Accordingly, only these 17 elements were included in the multi-element method.

Quantitation was performed via external calibration (Table S8) and Ge, Rh, In, and Re were used as internal standards. Before each measurement, the instrument’s optimization and calibration were performed using a tuning solution (containing Li, Y, and Tl; see Table S8) to ensure maximum sensitivity over the entire mass range. At the same time, the oxide and doubly charged ratios were controlled. Additional instrument conditions are listed in Table S10. Method validation was performed in previous studies using the same ICP-MS [16,21].

### 3.4. Calculation of Element Concentrations and Statistical Analysis

The calculation of the element concentrations of the walnut mixtures was performed with Microsoft Excel 2019 (Microsoft Corporation, Redmond, WA, USA) using the 17 elements relevant for origin classification. The signals of the elements were related to the internal standard after blind value deduction and, afterwards, the element concentration was calculated using the external calibration range. The Unscrambler (version 11.0, CAMO Analytics) was used for the generation of the principal component analysis (PCA) models. The 206 original samples were used for the creation of different classification and regression models using R software (version 4.1.1), RStudio (2022.07.1), and various R packages. All classification and regression models in combination with the packages that were used in this study are summarized in Table 1. Nine different mixing ratios were calculated (90/10, 80/20, 70/30, 60/40, 50/50, 40/60, 30/70, 20/80, and 10/90) using all 206 walnut samples, resulting in 21,115 possible mixtures for every mixing ratio after reducing the sample size by removing mixtures from the same origin. All calculated mixtures were tested with different classification and regression models to figure out which one fitted best for the data. An overview can be found in Table 2 in Section 2.3. Additionally, all measured mixtures were evaluated with the same models for validation purposes.

Approach I includes one-class classification models using the pure samples of each country individually. The related mixtures were used as a test set. When a mixture was assigned to the model with pure samples, the sample was not detected as a mixture. In reverse, when a mixture was not assigned to the model containing only pure samples, the sample was successfully identified as mixture.

Approach II works with two-class classification models for each individual country. One class contains the pure samples of each country and the second class contains the corresponding mixtures of the country. The model uses an internal cross-validation and therefore the test set contains the same two groups. The classification was successful, when the pure samples and the mixtures were allocated to their own class.

In approach III two-class classification models were used, one class includes pure samples of one country and the other class contains the pure samples of all other countries. The test set contains the mixtures of the associated country. The classification was successful, when a mixture was classified as a sample from the other countries. When a mixture was classified as an “unadulterated sample” from the respective country the assignment was counted as false.

In addition, approach IV and V are dealing with classification and regression models where the calculated data was used for model creation. The calculated mixtures, which contained the same samples in the same mixing ratios as the measured mixtures, were excluded from model generation to avoid overfitting. The measured walnut mixtures were used as a test set to predict the exact mixing ratio. Additionally, the previously excluded calculated mixtures were also used as an independent test set. The conformity of the results was then evaluated.

## 4. Conclusions

High-resolution and sensitive methods are usually required to detect food fraud. Elemental profiling offers a number of advantages, such as low detection limits and invariability of analytes, and promising studies on the determination of food origin can also be found in the literature. So far, we are not aware of any study dealing with the analysis of mixtures from different origins using ICP-MS. The present study investigated whether an element profiling approach is suitable for the classification of mixtures from different origins. In addition, it was examined whether the calculation of mixtures from measured pure samples can support an experimental measurement of the mixtures and thus reduce the overall measurement effort. Analyzing complex sample mixtures of different origins can increase the effort exponentially. Overall, the equipment is very sophisticated and expensive. Five different state-of-the-art evaluation approaches were considered, but, depending on the approach, they showed contradictory results.

(i) In the first approach, one-class classifiers were tested, which are often used to detect adulteration using spectroscopy data. Unadulterated samples were tested for model building. Both applied models (OC-SVM and OC-SIMCA) showed contrary results but neither model showed good accuracies for mixtures and pure samples together. Moreover, no comparability between measured and calculated mixtures was given.
(ii) In the second approach, two-class-classification models were applied, which led to better accuracies in the detection of measured mixtures. Therefore, the second approach can be used to identify mixtures using elemental data. A high agreement between the measured and calculated data was achieved for the Chinese, Swiss, French, and American mixtures.
(iii) A third approach was carried out, showing good classification accuracies for the measured mixtures even at lower adulteration levels (up to 100% for the Swiss model). Consequently, the third approach is also suitable for the detection of measured mixtures. Additionally, the results of the third approach showed high correlation between the measured and calculated data (in most cases only 10% points of difference) and very good classification accuracies for adulteration levels, from 60% for all countries (80–100%), except for the Chinese model with very good accuracies (100%) for an adulteration level of 70%. The highest agreement between the sample sets was obtained for the German and Italian mixtures at all adulteration levels.
(iv) The fourth and fifth approach, in which all calculated mixtures were used to build a nine-class classification and regression model, produced poorer results. In contrast to the other approaches, the subset of calculated mixtures showed better results than the measured mixtures. With such large data sets, it might be possible to improve the results between the measured and calculated data by increasing the sample size. Therefore, the first three approaches (I–III) are preferable in terms of applicability.

In summary, for elemental data, approaches II and III are best suited for the detection of measured mixtures of different origins. The proof of principle has shown that different evaluation approaches are useful to determine which approach is best suited for a particular data set. Approach III showed that measured mixtures can be recognized even at low levels of adulteration (i.e., 100% accuracy for the Swiss model, even at 20% adulteration). The approaches with two-class classification models provided the most promising results for the elemental data used in this study. Measured mixtures can be predicted with both the second and third approaches using elemental data. Although the calculated and measured mixtures of walnuts from different origins are only comparable to a limited extent, a combination of the two approaches can be useful. By using the models based on calculated data in approach II, suspect samples can be analyzed and predicted in routine analysis. Depending on the country, the models of the third approach can then be used to validate the result.

## Figures and Tables

**Table 1:** Summary of the different data pretreatment methods, classification models, and regression models in combination with the packages used in this study.

Model	Package (Version)
one class classification	
one class—support vector machine, OC-SVM	e1071 (1.7-11, CRAN) [28]
one class—soft independent modelling of class analogies, OC-SIMCA	mdatools (0.14.1, CRAN) [29]
classification	
support vector machine, SVM	mlr3 (0.13.4, CRAN) [30], e1071 (1.7-11, CRAN)
linear discriminant analysis, LDA	mlr3 (0.13.4, CRAN), MASS (7.3-60, CRAN) [31]
random forest classification, RF-C	mlr3 (0.13.4, CRAN), ranger (0.14.1, CRAN) [32]
regression	
support vector regression, SVR	mlr3 (0.13.4, CRAN), e1071 (1.7-11, CRAN)
partial least squares regression, PLSR	mlr3 (0.13.4, CRAN), pls (2.8-1, CRAN) [33]
random forest regression, RF-R	mlr3 (0.13.4, CRAN), ranger (0.14.1, CRAN)
data pretreatment	
no data pretreatment, centering (mean) and scaling (standard deviation), centering (median) and scaling (interquartile range), decadic logarithm (log10)	

**Table 2:** Overview of the different evaluation approaches to detect adulteration of walnut samples.

Approach	Type	Training Set	Test Set	Positive	False
I	one-class classification	pure samples of one country	all mixtures from the specific country	a mixture was not assigned to the training set	a mixture was assigned to the training set
II	classification	pure samples of one country vs. all mixtures from the specific country	pure samples of one country vs. all mixtures from the specific country	the pure samples and the mixtures were allocated to their own class	the pure samples and the mixtures were allocated to the opposite class
III	classification	pure samples of one country vs. all other countries	all mixtures from the specific country	a mixture was classified as a sample from the other countries	a mixture was classified as an “unadulterated sample” from the respective country
IV	classification	calculated mixtures in all adulteration levels	all mixtures from the specific country	a mixture was assigned to the right adulteration level	a mixture was assigned to the wrong adulteration level
V	regression	calculated mixtures in all adulteration levels	all mixtures from the specific country	a mixture was assigned to the right adulteration level	a mixture was assigned to the wrong adulteration level

**Table 3:** Overview of the classification accuracies achieved with the different evaluation approaches (I–III), accuracies are given in %. Data pretreatment labeling as follows: * none, ^#^ log10, ^0^ mean and standard deviation, ¯ median and interquartile range.

Approach	Samples	China [%]	Switzerland [%]	Germany [%]	France [%]	Italy [%]	US [%]
approach I OC-SVM	pure samples	66.7	54.8	53.1	54.0	48.5	46.7
	measured mixtures	96.7 *	98.9 *	92.2 ^#^	86.8 *	73.3 *	98.9 *
	calculated mixtures	64.8 ¯	71.1 ¯	47.8 ^#^	54.0 *	58.9 ^0^	65.6 ¯
approach I OC-SIMCA	pure samples calibration	100	93.5	91.8	93.7	100	100
	pure samples validation	73.3	83.9	86.0	82.5	72.7	53.3
	measured mixtures	62.6	81.1 ^#^	56.7 ^#^	64.8 ^#^	38.9 ^#^	37.8 ^#^
	calculated mixtures	37.4 ^0^	36.7 ^#^	32.2 ^#^	34.1 ^#^	28.9 ^0^	26.7 ^0^
approach II RF (variant I)	measured mixtures	95.0 *	95.6 ^0^	86.5 ^0^	91.3 *	85.3 *	94.5 ^#^
	calculated mixtures	85.5 *	80.7 ^0^	68.7 ^0^	83.0 *	75.2 *	85.7 ^#^
approach II RF (variant II)	measured mixtures	85.5 *	80.7 ^0^	68.7 ^0^	83.0 *	75.2 *	85.7 ^#^
	calculated mixtures	100 *	97.8 ^0^	85.6 ^0^	81.3 *	96.7 *	96.0 ^#^
approach III SVM (variant I)	measured mixtures	63.7 ^#^	95.6 ^#^	70.0 ^#^	80.2 ^#^	63.3 ^#^	87.8 ^#^
	calculated mixtures	47.3 ^#^	66.7 ^#^	71.1 ^#^	65.9 ^#^	63.3 ^#^	72.2 ^#^

**Table 4:** Summary for the accuracy of the detected adulteration per adulteration level in approach III. Comparison of the sample set A (calculated mixtures) and sample set B (measured mixtures) for the German classification model.

Adulteration [%]	Accuracy Measured Data [%]	Accuracy Calculated Data [%]	Difference in Accuracy [%pt]
10	40.0	30.0	10.0
20	40.0	50.0	10.0
30	50.0	50.0	0.00
40	60.0	40.0	20.0
50	80.0	70.0	10.0
60	100	100	0.00
70	90.0	100	10.0
80	100	100	0.00
90	90.0	100	10.0

## Data Availability

Data set available on request from the authors.

## Supplementary Materials

The following supporting information can be downloaded at: https://www.mdpi.com/article/10.3390/molecules29143350/s1, Table S1: Formulas for calculation of possible mixing ratios for walnut mixtures depending on the amount of pure samples and nine possible mixing ratios used in this study; Table S2: Results of different classification models with different data pretreatment methods for the six-class classification model containing sample set C (pure samples); Table S3: Model parameters of the different approaches. When one parameter has multiple values, different settings per country were used; Table S4: Best results of the classification models for approach III with model parameters; Table S5: Summary for the accuracy of the detected adulteration per adulteration level for the SVM models. Comparison of the measured (sample set B) and calculated mixtures (sample set A); Table S6: Prediction accuracy of the French classification models as well as the measured (sample set B) and calculated mixtures (sample set A) predicted with the models, no data pretreatment; Table S7: RMSE and R2 of the French regression models as well as the measured (sample set B) and calculated mixtures (sample set A) predicted with the models, no data pretreatment; Table S8: Overview of chemicals and solutions used in this study; Table S9: Overview of the concentration of various elements in the certificated reference material (DLA ptSU08) and the concentration analyzed using the ICP-MS of this study; Table S10: Instrument conditions and measurement parameters for the Element2 HR-ICP-MS used in this study; Figure S1: Confusion matrixes for the classification models (RF) of German, Chinese, American and Italian walnut samples, each in comparison with all associated walnut mixtures, showing the accuracies in %. The left side shows measured walnut mixtures (sample set B), the right side calculated walnut mixtures (sample set A); Figure S2: Confusion matrixes for the classification models (SVM) of French, Italian and Swiss walnut samples, each in comparison with all other pure walnut samples (sample set C), showing the accuracies in %; Figure S3: Confusion matrix of the measured mixtures (sample set B) for classification of the different mixing ratios from the French walnut samples. Random forest model, no data pretreatment; Figure S4: Confusion matrix of the calculated mixtures (sample set A) for classification of the different mixing ratios from the French walnut samples. Random forest model, no data pretreatment.
