# Supplementary material for: Elemental Profiling for the Detection of Food Mixtures: A Proof of Principle Study on the Detection of Mixed Walnut Origins Using Measured and Calculated Data
Source: Molecules. 2024 Jul 17;29(14):3350. doi: 10.3390/molecules29143350 (PMC11279845; doi:10.3390/molecules29143350)
Supplement: Supplementary file 1 [file molecules-29-03350-s001.zip › molecules-3113704-supplementary.pdf]

Supporting Information

Elemental profiling for the detection of food mixtures: A proof of principle study on the detection of mixed walnut origins using measured and calculated data

Marie-Sophie Müller[1], Esra Erçetin[1], Lina Cvancar[1], Marie Oest[1] and Markus Fischer [1*]

[1] Hamburg School of Food Science, Institute of Food Chemistry, University of Hamburg, Grindelallee 117, 20146 Hamburg, Germany; mariesophie.mueller@uni-hamburg.de (M.-S.M.), esr.ercetin@gmail.com (E.E.), lina.cvancar@uni-hamburg.de (L.C.), marie.oest@uni-hamburg.de (M.O.), markus.fischer@uni-hamburg.de (M.F.)

* Correspondence: Markus Fischer, Hamburg School of Food Science, Grindelallee 117, 20146 Hamburg. Phone: +49-40-428384357/59. E-Mail: markus.fischer@uni-hamburg.de

Table S1. Formulas for calculation of possible mixing ratios for walnut mixtures depending on the amount of pure samples and nine possible mixing ratios used in this study.

topic	formula for calculation	possible combinations
pure samples	n	206
combinations for one mixing ratio for all countries	$\frac{n^2 - n}{2}$	21115
combinations for one mixing ratio of the same country	$\frac{n_1^2 - n_1}{2} + \frac{n_2^2 - n_2}{2} + [\ldots]$	4332
reducing combinations by mixtures of the same country	21115 - 4332	16783
number of possible mixing ratios used in this study	m	9
combinations for all mixing ratio for all countries except mixtures of the same country	$m \frac{n^2 - n}{2}$	151047

Table S2. Results of different classification models with different data pretreatment methods for the six-class classification model containing sample set C (pure samples).

data pretreatment	model accuracy [%]	model parameters
	**SVM**	
none	73.1	radial kernel, cost = 7, gamma = 0.04
mean & standard deviation	73.1	radial kernel, cost = 7, gamma = 0.04
median & interquartile range	73.1	radial kernel, cost = 7, gamma = 0.04
decadic logarithm	78.4	radial kernel, cost = 1, gamma = 0.07
	**RF**	
none	73.3	importance = permutation, trees = 750, mtry = 9
mean & standard deviation	73.3	importance = permutation, trees = 750, mtry = 9
median & interquartile range	73.3	importance = permutation, trees = 750, mtry = 9
decadic logarithm	73.9	importance = none, trees = 1000, mtry = 9
	**LDA**	
none	68.0	method = moment, predict.method = plug-in
mean & standard deviation	68.0	method = moment, predict.method = plug-in
median & interquartile range	68.0	method = moment, predict.method = plug-in
decadic logarithm	70.5	method = T, predict.method = predictive

Table S3. Model parameters of the different approaches. When one parameter has multiple values, different settings per country were used.

approach	model	parameters
I	OC-SVM	radial kernel, gamma = 0.059, nu = 0.5
	OC-SIMCA	limits = ddmoments, alpha = 0.05, gamma 0.01
II	RF	importance = permutation, impurity, impurity correction, none; trees = 500, 750, 10000; mtry = 9, 17
III	SVM	radial kernel, cost = 1,4, 7, 10; gamma = 0.01, 0.04, 0.07, 0.1
IV	RF	trees = 500, splitrule = gini
V	RF	trees = 500, splitrule = variance

Table S4. Best results of the classification models for approach III with model parameters.

country	model accuracy [%]	model parameters
China	99.3	SVM, radial kernel, cost = 7, gamma = 0.04
Switzerland	94.8	SVM, radial kernel, cost = 1, gamma = 0.1
Germany	85.6	SVM, radial kernel, cost = 1, gamma = 0.07
France	88.4	SVM, radial kernel, cost = 10, gamma = 0.04
Italy	95.2	SVM, radial kernel, cost = 7, gamma = 0.04
US	95.6	SVM, radial kernel, cost = 7, gamma = 0.04

Table S5. Summary for the accuracy of the detected adulteration per adulteration level for the SVM models. Comparison of the measured (sample set B) and calculated mixtures (sample set A).

adulteration [%]	accuracy measured data [%]	accuracy calculated data [%]	difference in accuracy [%pt]
	**China**		
10	40.0	0.00	40.0
20	0.00	0.00	0.00
30	80.0	00.0	80.0
40	0.00	40.0	40.0
50	90.0	30.0	60.0
60	60.0	70.0	10.0
70	100	90.0	10.0
80	100	100	0.00
90	100	100	0.00
	**Switzerland**		
10	80.0	20.0	60.0
20	100	40.0	60.0
30	100	50.0	50.0
40	100	40.0	60.0
50	70.0	50.0	20.0
60	100	100	0.00
70	100	100	0.00
80	100	100	0.00
90	100	100	0.00
	**France**		
10	40.0	10.0	30.0
20	100	40.0	60.0
30	50.0	50.0	0.00
40	100	40.0	60.0
50	80.0	70.0	10.0
60	80.0	80.0	0.00
70	80.0	100	20.0
80	100	100	0.00
90	90.0	100	10.0
	**Italy**		
10	10.0	0.00	10.0
20	30.0	10.0	20.0
30	50.0	30.0	20.0
40	80.0	80.0	0.00
50	70.0	70.0	0.00
60	80.0	80.0	0.00
70	90.0	100	10.0
80	80.0	100	20.0
90	80.0	100	20.0

adulteration [%]	accuracy measured data [%]	accuracy calculated data [%]	difference in accuracy [%pt]
		**US**	
10	30.0	30.0	0.00
20	80.0	30.0	50.0
30	90.0	40.0	50.0
40	100	80.0	20.0
50	100	70.0	30.0
60	100	100	0.00
70	100	100	00.0
80	100	100	00.0
90	100	100	00.0

Table S6. Prediction accuracy of the French classification models as well as the measured (sample set B) and calculated mixtures (sample set A) predicted with the models, no data pretreatment.

**classification model**	**accuracy of the model [%]**	**accuracy calculated data [%]**	**accuracy measured data [%]**
SVM	56.1	51.7	12.1
LDA	17.9	17.6	16.5
RF-C	68.1	85.7	27.5

Table S7. RMSE and R^2 of the French regression models as well as the measured (sample set B) and calculated mixtures (sample set A) predicted with the models, no data pretreatment.

regression model	model	calculated data	measured data
SVR	RMSE: 5.45%<br>R^2: 0.955	RMSE: 5.39%<br>R^2: 0.956	RMSE: 23.0%<br>R^2: 0.200
PLSR	RMSE: 25.0%<br>R^2: 0.0590	RMSE: 24.4%<br>R^2: 0.108	RMSE: 24.5%<br>R^2: 0.0964
RF-R	RMSE: 5.11%<br>R^2: 0.961	RMSE: 5.19%<br>R^2: 0.959	RMSE: 19.9%<br>R^2: 0.406

Table S8. Overview of chemicals and solutions used in this study.

Reagent	Comment	Manufacturer
water, ultrapure	> 18 MΩ	Direct-Q purifying system, Merck Millipore Inc., (Billerica, MA, USA)
nitric acid (HNO_3)	ROTIPURAN Supra, 69 %, *v/v*	Carl Roth GmbH & Co. KG (Karlsruhe, Germany)
hydrogen peroxide (H_2O_2)	suprapur, 30 %, v/v	Merck KGaA (Darmstadt, Germany)
multi-elemental standard solutions	- 10 mg/L Li, Na, Mg, Al, K, V, Cr, Mn, Co, Ni, Cu, Ga, Rb, Sr, Mo, Ag, Cd, Te, Ba, Tl, Pb, Bi and U (used in a range from 0.001 - 1000 µg/L)<br>- 100 mg/L Be, B, Fe, Zn, As, and Se (used in a range from 0.01 - 10.000 µg/L)<br>- 1,000 mg/L Ca (used in a range from 0.1 - 100.000 µg/L)	Merck KGaA (Darmstadt, Germany)
	1 g/L Ge, Rh, In, and Re (used in 100 µg/L)	Inorganic Ventures Inc. (Christiansburg, VA, USA)
reference material	RM 2020-SU8	DLA-Proficiency Tests GmbH (Oering, Germany)
argon	≥99.999 %	Sauerstoffwerk Steinfurt E. Howe GmbH & CO. KG (Steinfurt, Germany)

Table S9. Overview of the concentration of various elements in the certificated reference material (DLA ptSU08) and the concentration analyzed using the ICP-MS of this study.

element	concentration reference material [mg/kg]	concentration measured data [mg/kg]
As	0.230 ± 0.0212	0.341 ± 0.0170
B	0.681 ± 0.294	0.798 ± 0.147
Ba	0.949 ± 0.0708	0.924 ± 0.0021
Ca	10300 ± 1110	9253 ± 94.05
Cd	0.0757 ± 0.00994	0.0910 ± 0.00976
Cr	1.09 ± 0.0797	1.01 ± 0.0424
Cu	0.444 ± 0.0499	0.463 ± 0.0276
Fe	2.16 ± 0.537	1.79 ± 0.156
K	8800 ± 528	8229 ± 313
Mg	735 ± 51.1	669 ± 11.3
Mn	0.321 ± 0.0313	0.394 ± 0.0028
Mo	0.297 ± 0.0542	0.301 ± 0.0064
Na	2040 ± 188	2277 ± 9.90
Pb	0.194 ± 0.0191	0.190 ± 0.0064
Se	0.761 ± 0.180	0.962 ± 0.067
Zn	36.9 ± 4.70	33.1 ± 0.919

Table S10. Instrument conditions and measurement parameters for the Element2 HR-ICP-MS used in this study.

**forward power (W)**	1225	
**plasma gas flow rate (L/min)**	16.0	
**auxiliary gas flow rate (L/min)**	0.70	
**nebulizer gas flow rate (L/min)**	1.10	
**sample uptake (μL/min)**	200	
**cones**	Ni	
**nebulizer**	MicroMistTM	
**number of acquisition replica**	3	
**tuning**	doubly charged ratio: Ba^{++}/Ba^{+}<br>oxide ratio: BaO^{+}/Ba^{+}	
**acquisition mode**	E-Scan	
**resolution**	Low<br>(300mΔm-1)	Medium<br>(4000mΔm-1)
**selected isotopes for analytes**	11B, 85Rb, 88Sr, 95Mo, 125Te, 137Ba, 205Tl	24Mg, 27Al, 44Ca, 55Mn, 56Fe, 59Co, 60Ni, 63Cu, 66Zn, 71Ga
**selected isotopes for internal standard**	72Ge, 103Rh, 115In, 185Re	72Ge, 103Rh, 115In
**correction equations**	115In = 115In-0.0149·Sn118	115In = 115In-0.0149·Sn118

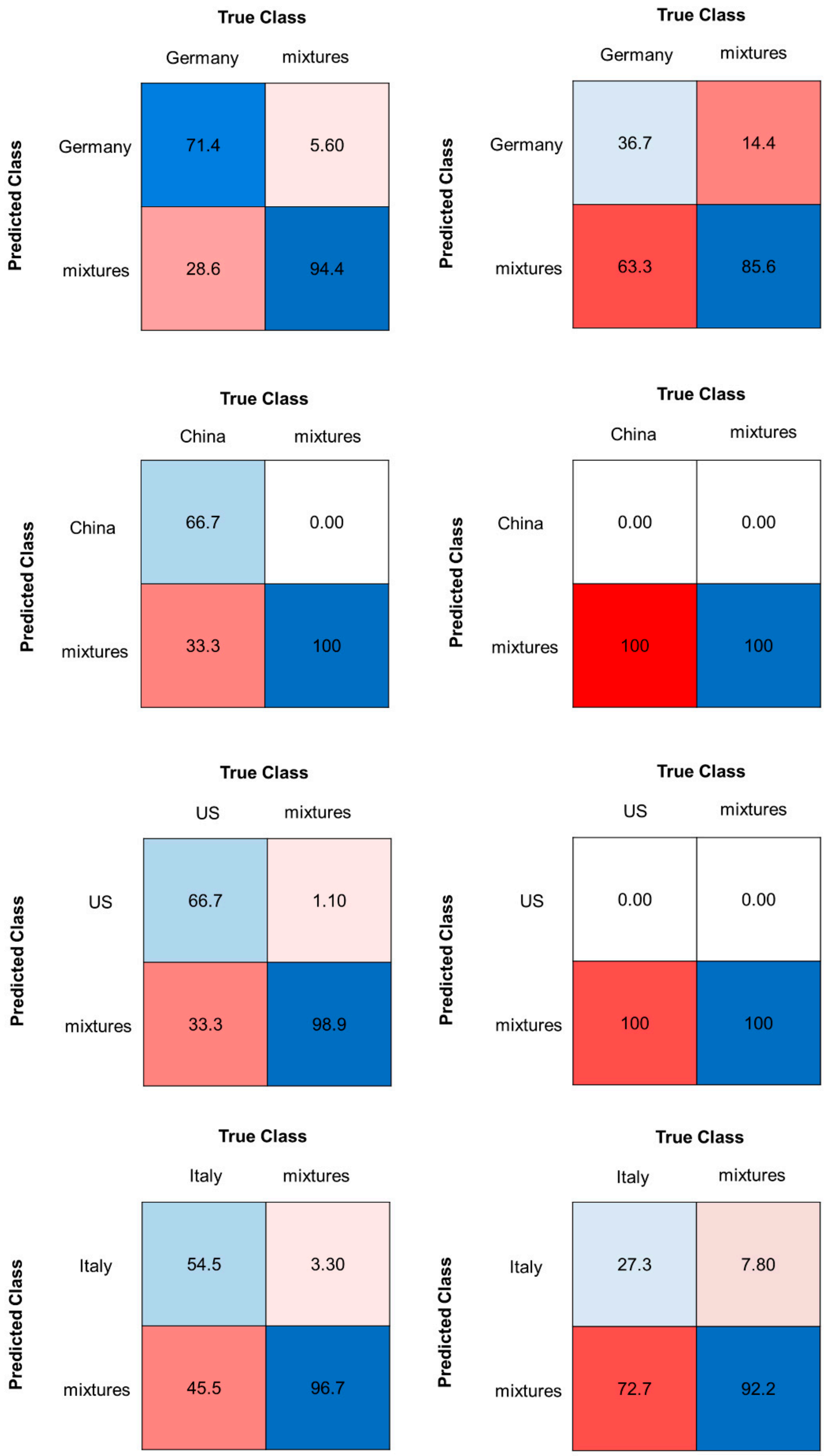

Figure S1. Confusion matrixes for the classification models (RF) of German, Chinese, American and Italian walnut samples, each in comparison with all associated walnut mixtures, showing the accuracies in %. The left side shows measured walnut mixtures (sample set B), the right side calculated walnut mixtures (sample set A).

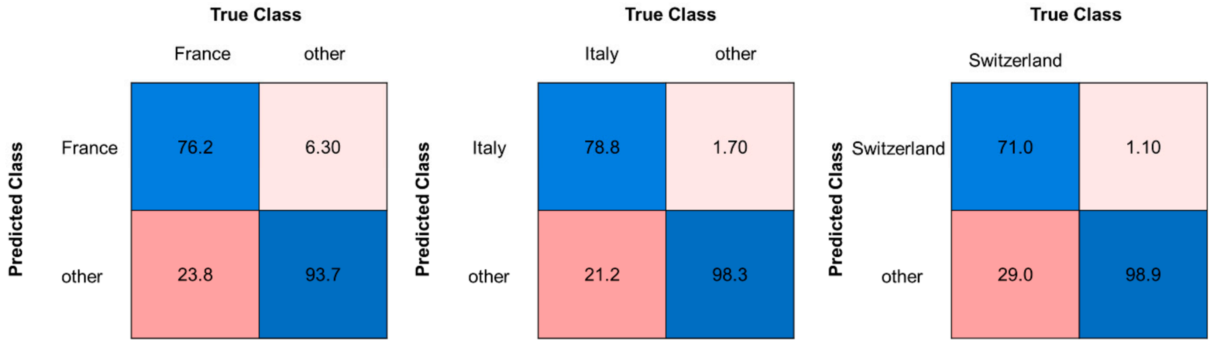

Figure S2. Confusion matrixes for the classification models (SVM) of French, Italian and Swiss walnut samples, each in comparison with all other pure walnut samples (sample set C), showing the accuracies in %.

real adulteration [%] (columns); **predicted adulteration [%]** (rows)

	10	20	30	40	50	60	70	80	90
10	0	0	0	0	0	0	0	0	0
20	3	3	2	0	0	0	1	0	0
30	3	2	3	1	1	0	1	0	0
40	1	3	2	5	4	1	2	1	0
50	2	2	3	4	1	2	2	0	1
60	0	0	0	0	2	5	2	2	1
70	0	0	0	0	2	1	2	2	3
80	1	0	0	0	0	0	0	5	4
90	0	0	0	0	0	1	1	0	1
**accuracy [%]**	0.00	30.0	30.0	50.0	10.0	50.0	20.0	50.0	10.0

Figure S3. Confusion matrix of the measured mixtures (sample set B) for classification of the different mixing ratios from the French walnut samples. Random forest model, no data pretreatment.

predicted adulteration [%] \ real adulteration [%]	10	20	30	40	50	60	70	80	90
10	10	1	0	0	0	0	0	0	0
20	0	9	1	1	0	0	0	0	0
30	0	0	9	2	0	0	0	0	0
40	0	0	0	6	1	1	0	0	0
50	0	0	0	1	7	0	0	0	0
60	0	0	0	0	2	9	1	0	0
70	0	0	0	0	0	0	10	0	0
80	0	0	0	0	0	0	0	8	0
90	0	0	0	0	0	0	0	2	10
**accuracy [%]**	0.00	30.0	30.0	50.0	10.0	50.0	20.0	50.0	10.0

Figure S4. Confusion matrix of the calculated mixtures (sample set A) for classification of the different mixing ratios from the French walnut samples. Random forest model, no data pretreatment.
